# Physical Therapy for Gait, Balance, and Cognition in Individuals with Cognitive Impairment: A Retrospective Analysis

**DOI:** 10.1155/2020/8861004

**Published:** 2020-11-03

**Authors:** Jason Longhurst, Jason Phan, Elbert Chen, Steven Jackson, Merrill R. Landers

**Affiliations:** ^1^Department of Neurorehabilitation, Cleveland Clinic Lou Ruvo Center for Brain Health, 888 West Bonneville, Las Vegas, Nevada, USA 702-483-6032; ^2^Department of Physical Therapy, University of Nevada, Las Vegas, USA; ^3^Department of Physical Therapy, University of Nevada, Las Vegas, 4505 Maryland Parkway, Box 453029, Las Vegas, Nevada, USA 702-895-1377

## Abstract

**Objectives:**

The purpose of this study was to determine if a pragmatic physical therapy (PT) program was associated with improved cognition, gait, and balance in individuals with cognitive impairment. This study investigated these associations for individuals with Alzheimer disease (AD), vascular dementia (VaD), dementia with Lewy bodies (DLB), and mild cognitive impairment (MCI) in order to better characterize outcomes to PT for each diagnostic group.

**Methods:**

Data before and after one month of physical therapy were extracted from patient records (67 with AD, 34 with VaD, 35 with DLB, and 37 with MCI). The mean number of PT sessions over a month was 3.4 (±1.8). Outcomes covered the domains of gait, balance, and cognition with multiple outcomes used to measure different constructs within the balance and gait domains.

**Results:**

All groups showed improvements in balance and at least one gait outcome measure. Those with MCI improved in every measure of gait and balance performance. Lastly, cognition as measured by Montreal Cognitive Assessment improved in individuals in the AD, VaD, and MCI groups.

**Conclusion:**

While this retrospective analysis is not appropriate for causal inference, results of one month of physical therapy were associated with decreases in gait, balance, and cognitive impairment in individuals with AD, VaD, DLB<, and MCI. *Clinical Implications*. While physical therapy is not typically a primary treatment strategy for individuals with cognitive impairment, the results of this study are consistent with the literature that demonstrates improvement from physical therapy for other neurodegenerative diseases. Further clinical and research exploration for physical therapy as a primary treatment strategy in these populations is warranted.

## 1. Introduction

Between 2012 and 2050, the population aged 65 years and older within the United States is expected to nearly double from 43.1 million to 83.7 million [1]. This growth will have wide-ranging implications for healthcare systems and its management of chronic diseases. Of particular concern are disorders resulting in cognitive impairment (CI) including Alzheimer disease (AD), vascular dementia (VaD), dementia with Lewy bodies (DLB), and mild cognitive impairment (MCI). Because of their progressive nature, these disorders can result in the loss of independence which strains individuals, families, and society [2].

Dementia is an umbrella term for a broad range of cognitive symptoms that cause functional impairment [3]. It encompasses a variety of subtypes that are categorized based on disease timing, severity of cognitive symptoms, and other specific diagnostic criteria [3]. With a prevalence of 5.3 million cases in the U.S. in 2015, AD is the most common cause of dementia in older adults and is the most common neurodegenerative disease [4]. It is characterized by beta-amyloid and phosphorylated-tau pathology [5]. In comparison, DLB is the second most diagnosed type of progressive dementia, and it is characterized by a primary synucleinopathy that causes protein deposits, called Lewy bodies, in the brain [3]. The presence of visual hallucinations and spontaneous motor features of parkinsonism makes DLB distinguishable from other dementias [6]. VaD is another type of dementia that is caused by vascular events in the brain [7]. MCI, a potential precursor to dementia, is marked by slight, but detectable, decline in cognitive abilities not reaching the threshold for dementia diagnosis.

Another contributor to functional decline in persons with CI is motor impairment [8–10]. These motor impairments have received less treatment and research attention compared to the more prominent cognitive impairments. Subsequently, while physical activity programs for the prevention of and management of CI have begun to be implemented, physical therapy (PT) to address the motor impairment has not been considered a primary treatment strategy for individuals with CI [11, 12]. However, evidence supports that exercise can mitigate some of the cognitive and motor impairment [8, 9]. Hence, there is a need to further explore the most effective circumstances and settings for providing these types of interventions to individuals with CI disorders.

Gait and balance dysfunctions are common in people with dementia [10]. Specifically, individuals with dementia exhibited slower gait speeds compared to healthy controls, and more severe dementia was associated with more severe declines in gait [8, 9]. Recognizing and addressing this connection are crucial as declining gait characteristics have been correlated with decreased survival and independence in older adults [13]. Another important motor impairment in individuals with CI is impaired balance which, combined with the gait deficits, significantly increases risk for falls [14–16]. Approximately 60% of individuals with CI fall annually, twice as frequently as their cognitively intact counterparts [17, 18]. Individuals with CI have higher rates of mortality and institutionalization postfall [19, 20] and are more likely to have falls resulting in injuries, with up to a threefold increase in hip fracture incidence over individuals without CI [19]. Falls with resultant injuries have been shown to warrant extensive medical care including long-term hospitalization and rehabilitation which not only diminish the quality of life of the individual but also come with significant economic costs [21].

Because CI disorders are associated with loss of independence or future loss of independence, it is important to address potentially mitigable motor impairments which may exacerbate or hasten disability. Targeting motor impairments early may potentially prevent loss of function and also may delay the progression of CI [10, 22]. While evidence has suggested a connection between diminished mobility and diminished cognition, the opposite is also true: improving mobility has been shown to improve cognition. In fact, evidence suggests that improvements in the Six Minute Walk Test correlated with significantly less decline on the Mini-Mental State Examination in those with AD [23].

Currently, treatments for CI include both pharmacological and nonpharmacological interventions. Appropriate cognitive pharmacotherapy (e.g., cholinesterase inhibitors for AD and other memory enhancers) produces improvements in gait velocity, stride time, and fall risk [24]. Aerobic exercise has been shown to preserve mental speed and attention in individuals with AD [25]. Moreover, dual-task-based training can improve gait and balance in individuals with dementia [24, 26].

With all this evidence promoting the importance of mobility for individuals with CI, some medical doctors have started prescribing PT. [27] Referral to PT for the primary treatment of motor impairment associated with CI is not currently considered a first-line intervention because there is no consensus on its efficacy. In fact, although several studies have investigated this topic, it remains unclear whether individuals with CI will benefit from individualized PT, as opposed to the more commonly studied exercise programs, with respect to motor performance and fall risk prevention [28–30]. Therefore, the first aim of this study was to determine if a pragmatic individualized PT program over the course of four weeks is associated with decreases in gait and balance impairment in different diagnostic groups of individuals with CI. The intent was to investigate associations within each group of diagnoses rather than a between-group comparison. The second aim of this study was to determine if PT was also positively associated with cognition.

## 2. Methods

### 2.1. Design

A retrospective, pre- and post-PT cohort design was used in which balance and gait performance scores of individuals with CI disorders were extracted from medical records at the Cleveland Clinic Lou RuvoCenter for Brain Health (CCLRCBH) at the start and end of one month of PT. CCLRCBH is a comprehensive brain health facility in a large metropolitan area, consisting of 8 neurologists, 5 nurse practitioners and physician assistants, 4 physical therapists, 2 occupation therapists, a speech therapists, 4 neuropsychologists, 4 investigational researchers, and 4 social workers. CCLRCBH specializes in clinical management and investigational research in neurodegenerative diseases and related disorders. Specific demographic items that were extracted include sex, race, age, diagnoses, ICD 10 codes, assistive device usage, acetyl cholinesterase inhibitor use, and fall history in the 12 months prior to PT. None of the treating physical therapists were involved in the data extraction process. To determine what kind of treatment was offered, the total number of PT sessions was recorded, billing codes were analyzed, and treating therapists were interviewed annually regarding treatment structure and goals, as part of an effort to ensure treatment fidelity within the clinical facility. Treatment was organized into general categories (e.g., aerobic activity, strengthening, balance training, dual-task training, education, and functional training) and is detailed below.

### 2.2. Participants

**Figure 1:**
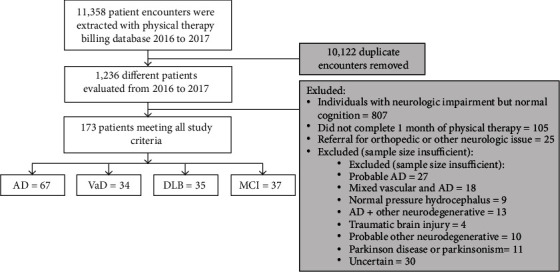
Data extraction flow diagram for individuals with Alzheimer disease (AD), vascular dementia (VaD), dementia with Lewy bodies (DLB), and mild cognitive impairment (MCI).

All individuals, ages 50 to 90, with an initial PT evaluation at CCLRCBH in 2016 and 2017 were identified from billing records. CCLRCBH considers PT to be an integral part of treatment for individuals with CI; therefore, individuals with CI are referred to PT by neurologist or nurse practitioners to address related motor impairments regardless of severity as a facility standard. Clinical diagnosis of disorders of cognition was completed by neurologists using specific sets of contemporary evidence-based criteria [5, 31–34]. For MCI and AD, the diagnostic guidelines from the 2011 National Institute on Aging-Alzheimer's Association workgroups were used. The diagnosis of VaD utilized the National Institute of Neurological Disorders–Canadian Stroke Network recommendations for diagnosis. For a diagnosis of DLB, the fourth consensus report of the DLB consortium was utilized. Inclusion criterion for this study was a referral to in-house PT for training and development of a comprehensive, individualized exercise program aimed at prevention and slowing of disease progression regardless severity and motor status. One month of PT is the typical duration of the initial prescription and is always followed by a reassessment. As the intent of this study was to investigate the effects of one month of physical therapy intervention, individuals were excluded if they did not complete one month of PT for any reason. Reasons for failing to complete one month of physical therapy were not collected. Individuals were also excluded if they were referred to PT for impairments apart from the standard protocol. This includes treatment of impairments such as vestibular dysfunction, amputation, significant lower extremity osteoarthritis, acute lower extremity surgery, lower extremity injury (fractures, strains, and sprains), or other neurologic disorders (e.g., traumatic brain injury, Parkinson disease). The original intent was to analyze the data for as many CI disorders that have sufficient numbers for analysis: subsequently CI disorders with small numbers were excluded (Figure 1). Data from 173 medical records were extracted, and, of those, 67 were classified as having AD, 34 as VaD, 35 as DLB, and 37 as MCI (Table 1 and Figure 1). The sample size was estimated post hoc using the “Mann-Whitney *U* or Wilcoxon Rank-Sum Tests” module in PASS 19.0.1 (NCSS, LLC. Kaysville, Utah, USA, http://ncss.com/software/pass). To detect an anticipated effect size of 0.85 between the pre- and post-Six Minute Walk Test (6MWT), a minimum of 25 participants were needed for each diagnostic group. The 6MWT was selected for sample size estimation as a primary focus of rehabilitation in this population is aerobic fitness, as it exerts both functional, cognitive, and neuroprotective effects for individuals with cognitive impairment [35–37]. The effect size was estimated based on clinical experience.

### 2.3. Outcome Measures

Outcome measure data from the following three domains (detailed below) were extracted from the medical record: cognition, gait, and balance. Balance and gait outcome measures were assessed by the treating physical therapists at initial evaluation and after 1 month of PT treatment at reassessment. Cognitive outcomes were assessed by neurologists at office visits prior to (average days prior to PT evaluation was 42.4 days ± 24.2) and immediately following physical therapy (average days prior to PT evaluation and following PT reassessment after 1 month of physical therapy were 42.4 days ± 24.2 and 41.7 ± 19.7, respectively). Since the study was retrospective, all assessors were technically blinded to the aims of the study. The minimal detectable change (MDC) and minimal clinically important differences (MCID) used in these analyses were from individuals with dementia when available. When the particular test did not have a MDC or MCID available in individuals with dementia, the value was taken for a population that most closely approximated the sample in this study.

As gait and balance are domains that are broad and contain multiple constructs that contribute to an individual's performance, multiple outcome measures were utilized in order to best characterize performance across these different constructs. Within the gait domain, measures were included that provided information regarding the following constructs: usual gait, gait adaptation, gait tolerance, functional gait, and dual-task gait. Within the balance domain, measures were included that provided information regarding the following balance constructs: anticipatory balance, reactive balance, sensory organization, and dynamic balance. A measure of fear of falling avoidance behavior was also included in the balance domain because the mitigation of downstream consequences of balance impairment was also another important part of the physical therapy treatment intervention.

#### 2.3.1. Gait

Scores from the following gait measures were included: Preferred Gait Speed (PGS) [38] (usual gait), Fast Gait Speed (FGS) [38] (gait adaptation) 6MWT [39] (gait tolerance), Timed Up and Go Test (TUG) [40] (functional gait), and Timed Up and Go Cognitive Test (TUGcog) [41] (dual-task gait). Both the PGS and FGS have excellent reliability in elderly individuals (ICC = 0.94 and 0.96, respectively) [42]. The MDC for the PGS and FGS is 0.13 meters/second and 0.21 meters/second scale points for people with AD [43], while the MCID for the PGS is 0.06 meters/second in community dwelling older adults [44]. The 6MWT has excellent test-retest reliability (ICC = 0.982 − 0.987), interrater reliability (ICC = 0.97 − 0.99), and intrarater reliability (ICC = 0.76 − 0.9) for individuals with AD [43, 45]. Its MDC is 33.5 meters for people with AD [43], and its MCID is 20.0 meters in community dwelling older adults [44]. The TUG has excellent test-retest reliability (ICC = 0.987), intrarater reliability (ICC = 0.91), and interrater reliability (ICC = 0.92) for individuals with AD [43]. The MDC of the TUG in people with AD is 4.09 seconds [43]. The TUGcog was performed following the TUG and utilized a secondary cognitive task of serial backwards counting by 3. Individuals were instructed to perform both the motor and cognitive tasks as quickly and accurately as possible. The MDC for the TUGcog is 4.69 seconds in AD [46].

#### 2.3.2. Balance

Scores from the Mini Balance Evaluation Systems Test (MBT) [47] and the Five Times Sit-to-Stand Test (5STS) [48] were included to describe balance performance. The MBT measures postural, anticipatory, and reactive balance as well as sensory orientation and dynamic gait. The MBT has excellent interrater reliability (ICC = 0.98) and a MDC and MCID of 3.4 scale points in people with Parkinson disease [49–51]. Although it is typically used to measure functional lower limb strength, the 5STS is also considered a measure of anticipatory and dynamic balance in older adults [52]. The 5STS has excellent test-retest reliability for community-dwelling elderly (ICC = 0.957). It has an MDC of 2.73 seconds in people with AD [46, 53] and a MCID of 3.7 seconds in individuals with progressive neurologic conditions [54].Scores from the Modified Fear of Falling Avoidance Behavior Questionnaire (mFFABQ) were included to describe avoidance behavior due to fear of falling [55, 56]. The mFFABQ is a self-administered 14 item questionnaire score out 56 possible scale points. The mFFABQ has good overall test-retest reliability (ICC = 0.796) with a 90% MDC of 15.8 scale points in individuals with Parkinson's disease [56].

#### 2.3.3. Cognition

Cognition was measured using the Montreal Cognitive Assessment (MoCA) [57]. The MoCA was designed to differentiate MCI from dementia. Due to this, it has a higher ceiling than other general cognitive screening measures; however, as a result, it also has a floor effect in quantifying very severe cognitive impairment. This study utilized 2 different versions of the MoCA for the pre- and postassessments to avoid any learning effects influencing performance at the postassessment. The MoCA has been shown to have excellent test-retest reliability (correlation coefficient = 0.92) and excellent positive and negative predictive values for AD (89% and 100%, respectively) [57]. The MDC for the MoCA is 4 scale points, out of 30 possible scale points, for older adults [58], while the MCID is 2.15 scale points among adults after stroke [59].

### 2.4. Treatment Approach

Referral to the physical therapists from the neurologists at (blinded) consisted of instructions to evaluate and treat. Physical therapists then determined the most appropriate frequency of visits. Typically, the individuals were instructed to come to PT once per week (mean PT treatments = 3.4 ± 1.8) for one month. PT consisted of the following treatment parameters: aerobic activity (20-25 minutes), strengthening (15-20 minutes), and balance training (15-20 minutes). Because this was a pragmatic study, therapists had leeway to control the intensity and/or the exercise modality provided it fit within trial parameters. In addition, patients had some autonomy of choice for exercise modality. Cognitive and motor dual-tasking and functional training were incorporated into all three parts of the treatment program. To prevent practice effects, none of the exercises in the protocol were the same as the outcome measures. Education to individual and caregiver and a simplified home exercise program, consisting of simplified activities with in the same domains, were also included in each treatment session.  Table 2 contains a more detailed description of the PT treatment program.

### 2.5. Data Analysis

All analyses were conducted using SPSS 24.0 (IBM SPSS Statistics for Windows, Armonk, NY: IBM Corp) with *α* = 0.05. Missing values were imputed using the last observation carried forward method. Due to relatively small sample sizes, nonparametric analyses were conducted. Specifically, a two-sided Wilcoxan signed-rank test was used to compare the pre- and postscores on all of the variables for each of the pathologies. To correct for multiple comparisons, we used a Benjamini-Hochberg corrected *p* value for each of the analyses using an online calculator (https://egap.shinyapps.io/multiple-comparisons-app/). The pre- and postdifference scores on all of the variables were analyzed for improvement beyond the MDC values. The percentage of those who improved beyond the MDC was calculated for each outcome measure. Severity of cognitive impairment prior to beginning intervention was investigated for inclusion as a covariate (*r* > 0.40) but did not meet the threshold for inclusion in any of the analyses. To control for the potential confound of acetyl cholinesterase inhibitor use in improvements, those using and not using were compared for each diagnostic group using Wilcoxon signed-rank tests.

## 3. Results

### 3.1. Alzheimer Disease

Refer to Table 3 for a more detailed listing of pre- and post-PT outcome measure scores. Scores on the MoCA improved over the month of PT for those with AD (*p* = .009) with 20.8% improved beyond the MDC, and 30.2% improved beyond MCID. Although there was only 6.2% improvement in gait distance on the 6MWT, it was a statistically significant improvement (*p* = .003), and almost half improved beyond the MDC and MCID (46.2%). In addition, scores improved on the MBT (*p* = .003), the 5STS (*p* = .005), and the TUG (*p* = .003) with improvements beyond MDC 30.4%, 27.9%, and 14.1%, respectively. Additionally, 30.4% of individuals improved beyond MCID on the MBT, while 16.4% improved beyond MCID on the STS. Lastly, individuals reported less fear of falling avoidance behavior on the mFFABQ (*p* = .032). Overall, 76.1% of individuals with AD improved beyond the MCID for at least one measure of gait, balance, or cognition.

### 3.2. Vascular Dementia

Refer to Table 4 for a more detailed listing of pre- and post-PT outcome measure scores. In general, there were fewer outcome measures with statistically significant improvement for those with VaD. Scores on the MoCA improved over the month of PT for those with VaD (*p* = .045), but only 9.7% and 16.1% improved beyond the MDC and MCID, respectively. Scores also improved on the MBT (*p* = .045) with 29.2% improving beyond the MDC and MCID. Individuals with VaD were able to improve their 5STS (*p* = .045) with 44.8% and 41.4% improving beyond the MDC and MCID, respectively. The TUG (*p* = .045) also improved over the month of PT (*p* = .024) with 17.6% improving beyond the MDC. Overall, 67.6% of individuals with VaD improved beyond the MCID for at least one measure of gait, balance, or cognition.

### 3.3. Dementia with Lewy Bodies

Refer to Table 5 for a more detailed listing of pre- and post-PT outcome measure scores. As a group, those with DLB did not improve on the MoCA (*p* = .096) despite 29.0% improving beyond the MCID. There was improvement on the 6MWT (*p* = .024) with 41.7% improving beyond the MDC and MCID. Scores on the MBT (*p* = .005) and 5STS (*p* = .005) improved after a month of PT with 26.7% improving beyond MDC and MCID on the MBT. While on the STS, 43.8% improved beyond the MDC, and 40.6% improved beyond the MCID. In addition, the TUG (*p* = .005), TUGcog (*p* = .049, PGS (*p* = .005), and FGS (*p* = .005) all improved as well. Overall, 74.3% of individuals with DLB improved beyond the MCID for at least one measure of gait, balance, or cognition.

### 3.4. Mild Cognitive Impairment

Refer to Table 6 for a more detailed listing of pre- and post-PT outcome measure scores. Scores on the MoCA (*p* = .017) and MBT (*p* = .005) improved over the month of PT for individuals with MCI with 29.7% and 27.3% improving beyond the MCID, respectively. Scores on the 5STS (*p* = .006) also improved with 30.3% improving beyond the MDC and 27.3% improving beyond MCID. Improvements in TUG (*p* = .005) and TUGcog (*p* = .007) were also observed; however, only 8.1% improved on the TUG beyond the MDC. Performance on the PGS (p = .017) and FGS (p = .016) improved with MDC improvements ranging from 24.2% to 35.3%. Lastly, performance of the 6MWT (*p* = .025) improved with 31.0% improving beyond MDC and MCID. Overall, 81.1% of individuals with MCI improved beyond MCID for at least one measure of gait, balance, or cognition.

Across all diagnostic groups, there were no differences found between those who were using acetyl cholinesterase inhibitors and those who were not (*p*′s > .255), nor between those who had started acetyl cholinesterase inhibitors in prior 6 months and those who had not (*p*′s > .135), on any measure.

## 4. Discussion

The aims of this study were to investigate how one month of individualized PT affected cognition, balance, and gait in individuals with CI. Each group with CI showed significant improvement in at least two of the aforementioned domains. Collectively, it appears that multicomponent PT (aerobic activity, strengthening, and balance training) with a rather low treatment session frequency over one month (3.4 visits on average) and the implementation of an individualized home exercise program was sufficient to drive meaningful improvement across the four different CI diagnoses. In addition, it is interesting to note that all groups but DLB had statistically significant improvements in cognition. However, we caution interpretation of these results as the design (no control group) is not strong for causal inference, and we cannot rule out a learning effect on the outcome measures.

The MCI group appeared to benefit the most from PT as they improved in all three domains (gait, balance, and cognition) and in 8 of 9 outcome measures. The AD group showed improvements in all three domains, and the DLB group improved in gait and balance, but not cognition. Both AD and DLB groups improved in 7 of 9 outcomes, whereas the VaD group only improved in the balance and cognitive domains and in only 4 of the 9 outcomes across the domains. Since MCI is considered a symptomatic prodromal dementia, it is generally in the early stages and less severe cognitively. The mean MoCA scores of the four groups in this study support that notion. Taken together, the fact that the MCI group appeared to have more robust improvements may suggest that cognitive capacity may be important to potential improvements in balance and gait. These results parallel the findings of previous research which suggest that older adults with MCI are more likely to experience benefits from exercise compared to those with dementia [60]. Furthermore, our results are consistent with literature proposing that early treatment of gait and balance problems in individuals with CI may improve function and mobility [10]. These findings are especially important because they support research, which suggests that administering treatment during the preclinical phase may be more effective than waiting until symptoms arise [61]. Specifically with AD, the subtle pathophysiological changes are thought to occur at least one decade, if not several, before the clinical phase [62–64]. With many AD drug trials showing lackluster results in the later stages of disease, researchers are advocating further investigation into preclinical detection and treatment strategies as a way to mitigate or even prevent cognitive decline [62–64].

Balance was significantly improved in each of the four CI groups. All groups improved in both balance outcome measures. Interestingly, the VaD group improved the least overall despite initial hypotheses that it might have more potential for improvement than the AD and DLB groups due to higher baseline cognitive scores (Table 4). One possible explanation may be that the VaD group had higher fear of falling avoidance behavior overall which can result in reduced physical activity and in turn more impaired balance in elderly individuals [55].

While it has been previously demonstrated that exercise can benefit individuals with CI with respect to mobility, these findings have not been specifically observed in the context of PT. While previous studies using rehabilitation methods like those found in PT did show that exercise improved functional mobility in individuals with CI [28], other studies that prescribed multicomponent, balance, or functional training found improvement in their individuals' mobility [65–68]. Compared to a simple progressive exercise protocol, PT offers individualized, impairment-based care in which a movement expert assists individuals in achieving mobility goals using therapeutic exercise, patient education, feedback, a home exercise program, and appropriate rehabilitation technologies. These differences may explain why the individuals in this study were able to make significant improvement in cognition, balance, and gait measures within a short time (1 month) and with few treatment visits (3.4 ± 1.8).

Improvements were not as robust with the mFFABQ in which only the AD group experienced an improvement. Perhaps one month is a too short time for these individuals to gain balance confidence and decrease avoidance behavior due to a fear of falling. In support of this theory, a meta-analysis on six treatment programs for fear of falling in the elderly population showed that the best outcomes were reached after four months of intervention [69]. Because the current study only examined the effects of one month of intervention, it is likely that there was not enough time to improve fear of falling avoidance behavior across the majority of the groups. The meta-analysis also found that programs combining exercise and education were the most successful in reducing fear of falling [69]. Exercise and education were both key components of the PT intervention that was investigated in the current study; however, it is possible that the education was not retained well due to the individuals having CI. Overall, these findings suggest that either the one month of PT treatment was not long enough or it was not effectively designed to modify fear of falling avoidance behavior in individuals with CI.

Considering the progressive nature of these CI disorders, the findings of this study are noteworthy. With one month of PT, 75.1% of individuals experienced improvement beyond the MCID for motor or cognitive function. At this point, it is premature to make conclusions that PT should be a first-line therapy for individuals with CI. However, based on these preliminary findings, more rigorous study designs are certainly warranted. Future studies should investigate the dosing, duration, and frequency of PT and also experiment with extending the follow-up periods. Additionally, future research on this topic should utilize more robust study designs that allow for causal inference (e.g., designs with a control group).

It has been reported that acetyl cholinesterase inhibitors can influence physical outcomes in individuals with CI [70]. To investigate this as a potential contributor to the improvements seen in this study, acetyl cholinesterase inhibitor use was analyzed. No meaningful differences were found in any group between those who were using acetyl cholinesterase inhibitors and those who were not. Nor were there differences between those who started acetyl cholinesterase inhibitors in the last 6 months [70]. Though acetyl cholinesterase inhibitor use does not appear to influence these results, there are several other possible contributing factors. These factors include the effects of socialization, education, caregiver support, and learning to perform tasks with the existing function without achieving physiological change; however, the influence of each of these is generally a part of a structured rehabilitation program.

While the findings are promising, it is important to address the limitations of this study. First, it is a retrospective cohort study with no control group. Thus, results from this study should be interpreted with caution as this design is not appropriate for causal inference and we cannot rule out a learning effect on the outcome measures. Also, several of the nonsignificant results were trending in the right direction but were not statistically significant; thus, type II errors are probable when considering the relatively small sample sizes. This study utilized as one its outcomes the mFFABQ, a self-report measure of avoidance behavior due to fear of falling, which has not been established in dementia or CI. Self-report measures are generally not as reliable in patients with CI. As a result, the findings regarding this questionnaire should be interpreted with caution. While adherence to HEP was encouraged and an integral part of the intervention, adherence was not tracked. This study excluded participants who did not complete one month of PT, which may lead to potential bias in the results. It could be assumed that those individuals who continued and completed one month of PT were most likely those individuals who gained the greatest benefit from the intervention. There may be a subset of individuals that may have perceived less or no benefit that were excluded because they stopped attending the physical therapy. Additionally, this study was calibrated for the 6MWT and utilized fixed *α* and *β* which may have resulted in some outcome measures being underpowered and susceptible to type II error. Because of the multiple comparisons, there is also a potential for an increased risk in type I error; however, we conducted Benjamini-Hochberg corrections to mitigate this potential limitation. Lastly, the interventions were based on individual impairment instead of a standardized exercise program. This method can be seen as both a strength and a weakness as this impairment-based treatment is most consistent with clinical practice; on the other hand, it introduces more treatment variance than is typical of a well-controlled trial.

## 5. Conclusions

In conclusion, one month of low frequency, short duration, pragmatic PT addressing motor impairment and function was associated with decreases in gait, balance, and cognition impairment among individuals with AD, VaD, DLB, and MCI. Further clinical and research exploration for physical therapy as a primary treatment strategy in these populations is needed to demonstrate the efficacy in these populations.

## Figures and Tables

**Table 1:** Characteristics of the individuals with Alzheimer's disease, vascular dementia, dementia with Lewy bodies, and mild cognitive impairment (MCI).

	Alzheimer's disease *N* = 67	Vascular dementia *N* = 34	Dementia with Lewy bodies *N* = 35	MCI *N* = 37	Patients who did not complete 1 month of PT *N* = 105
Sex					
Male	32	18	24	18	50
Female	35	16	11	19	55
Race					
White	53	27	29	29	77
African American	3	1	3	3	10
Asian or Pacific Islander	1	1	1	2	3
Two or more	1	3	0	2	3
Missing	9	2	2	1	12
Assistive device					
None	47	18	21	28	86
Cane	6	3	6	7	3
Front wheel walker	4	5	3	1	5
Rollator	6	3	3	1	9
Wheelchair	1	3	0	0	1
Missing	3	2	2	0	1
Faller status					
Faller (>1 fall in last year)	37	22	22	19	61
Nonfallers	28	12	12	16	44
Missing	2	0	1	2	0
Miscellaneous					
Age (mean ± standard deviation)	78.8 ± 8.4	76.4 ± 9.3	77.0 ± 6.0	74.0 ± 9.0	76.0 ± 10.6
Number of physical therapy treatment sessions (mean ± standard deviation)	3.1 ± 1.8	3.5 ± 1.6	3.8 ± 4.0	3.6 ± 1.9	1.5 ± 0.9
Number of falls in the last year (mean ± standard deviation)	1.1 ± 1.0	2.0 ± 3.6	3.8 ± 8.7	3.1 ± 7.9	1.7 ± 2.8

**Table 2:** Overview of treatment program for individuals with memory impairment disorders.

*Aerobic activity* (20-25 minutes)	
(1) Treadmill, over ground gait, recumbent stepper (2) Goal: heart rate at 65-80% max zone or RPE: 13-15/20 (i) Patient's and caregivers were instructed to achieve one of the following three criteria as per CDC's physical activity guidelines for older and were provided with the CDC's physical activity guidelines: (a) Provided with heart rate chart and target zone (b) Can talk but not sing (c) Use RPE of 13-15/20 (3) Dual tasking: consistent utilization of a secondary task, either motor or cognitive (see below)	

*Strengthening* (15-20 minutes)	
Utilization of simple and/or functional strengthening activities based on the components of OTAGO program [71].	

*Balance training* (15-20 minutes)	
Training included incorporation of static, anticipatory, and reactive aspects of postural control; with application to functional tasks [72].	

*Utilization of dual-task (DT) training*	
Dual tasking was utilized during every type of intervention, modifying to the specific level of the individual and to the primary task.	

Examples of secondary cognitive tasks	Examples of secondary motor tasks
(1) Executive function (attention, visual scanning, switching, etc.) (2) Problem solving and planning (3) Working memory (short term memory, rehearsal, etc.)	(1) Functional—completion of ADLs/IADLs (dressing, cleaning, cooking, etc.) (2) Carrying or manipulating objects

*Educational interventions*	
(1) All sessions completed with individual and primary caregiver when available with time spent educating caregiver on appropriate cueing and engagement strategies (implicit learning, strength-based approach, etc.). (2) Education on cognitive benefits of exercise and recommendations for exercise as well as promotion of brain health related activities.	

*Functional training*	
Functional training oriented to the specific level and function of individual with extensive caregiver involvement in cueing and training strategies, including training with assistive device when appropriate.	

*Home exercise program*	
(1) Simplified and individualized home exercise program handouts designed for individuals with cognitive impairment and their caregiver (2) Home exercise program was encouraged to be completed 5 days each week and should include exercises from each domain. (3) Typical home program duration was 30-60 minutes of exercise	

**Table 3:** Pre- and posttest scores across the assessment battery for individuals diagnosed with Alzheimer's disease with Wilcoxon signed-rank *p* value and Benjamini-Hochberg adjusted *p* value.

	*N* = 67	Pre-mean (mean rank)	Post-mean (mean rank)	Effect size (power)	*p* value	Adjusted *p* value	% improved beyond MDC (MCID)
MoCA (scale points)	53	14.6 ± 5.2 (12.0)	15.8 ± 6.3 (21.1)	0.395 (80.6%)	.005	.009	20.8% (30.2%)
MBT (scale points)	56	17.6 ± 4.2 (5.0)	20.0 ± 4.2 (19.8)	0.842 (100%)	<.001	.003	30.4% (30.4%)
5STS (seconds)	61	17.0 ± 11.7 (23.93)	15.1 ± 10.0 (24.23)	0.325 (70.5%)	.002	.005	27.9% (16.4%)
TUG (seconds)	64	14.6 ± 9.9 (30.22)	13.4 ± 12.1 (20.18)	0.200 (35.1%)	.001	.003	14.1% (-)
TUGcog (seconds)	58	20.8 ± 12.2 (25.0)	19.7 ± 14.2 (20.1)	0.120 (14.6%)	.077	.087	19.0% (-)
PGS (meters/second)	64	0.86 ± 0.30 (23.7)	0.90 ± 0.30 (26.36)	0.152 (32.9%)	.025	.032	26.6% (39.1%)
FGS (meters/second)	62	1.32 ± 0.37 (20.2)	1.38 ± 0.42 (24.4)	0.225 (54.3%)	.091	.091	16.1% (-)
6MWT (meters)	39	319.1 ± 112.7 (9.1)	338.9 ± 118.3 (16.4)	0.509 (93.0%)	.001	.003	46.2% (46.2%)
FFABQmod (scale points)	51	16.4 ± 15.8 (16.2)	13.1 ± 14.5 (12.4)	0.347 (78.8%)	.022	.032	7.8% (-)

**Table 4:** Pre- and posttest scores across the assessment battery for individuals diagnosed with vascular dementia with Wilcoxon signed-rank *p* value and Benjamini-Hochberg adjusted *p* value.

	*N* = 34	Pre-mean (mean rank)	Post-mean (mean rank)	Effect size (power)	*p* value	Adjusted *p* value	% improved beyond MDC (MCID)
MoCA (scale points)	31	20.4 ± 5.3 (6.0)	21.5 ± 5.3 (9.6)	0.547 (83.8%)	.005	.045	9.7% (16.1%)
MBT (scale points)	24	17.6 ± 3.4 (5.5)	19.2 ± 3.7 (8.6)	0.593 (79.5%)	.013	.045	29.2% (29.2%)
5STS (seconds)	29	17.9 ± 10.4 (16.6)	15.7 ± 12.7 (12.8)	0.237 (23.4%)	.019	.045	44.8% (41.4%)
TUG (seconds)	34	20.6 ± 29.1 (15.3)	16.6 ± 16.6 (12.6)	0.225 (24.7%)	.020	.045	17.6% (-)
TUGcog (seconds)	26	19.4 ± 14.4 (14.3)	18.9 ± 19.7 (12.4)	0.056 (5.9%)	.316	.384	23.1% (-)
PGS (meters/second)	27	0.76 ± 0.67 (11.8)	0.85 ± 0.37 (17.3)	0.174 (14.0%)	.341	.384	33.3% (48.1%)
FGS (meters/second)	26	1.13 ± 0.46 (12.8)	1.29 ± 0.64 (13.8)	0.470 (63.5%)	.062	.093	30.8% (-)
6MWT (meters)	22	273.7 ± 147.0 (6.7)	294.5 ± 158.5 (7.7)	0.422 (47.2%)	.041	.074	31.8% (31.8%)
FFABQmod (scale points)	21	20.1 ± 17.6 (6.0)	17.5 ± 14.9 (4.8)	0.267 (21.4%)	.386	.386	9.5% (-)

**Table 5:** Pre- and posttest scores across the assessment battery for individuals diagnosed with dementia with Lewy bodies with Wilcoxon Signed-rank *p* value and Benjamini-Hochberg adjusted *p* value.

	*N* = 35	Pre-mean (mean rank)	Post-mean (mean rank)	Effect size (power)	*p* value	Adjusted *p* value	% improved beyond MDC (MCID)
MoCA (scale points)	31	16.9 ± 6.7 (8.9)	17.8 ± 6.9 (11.5)	0.263 (29.4%)	.085	.096	19.4% (29.0%)
MBT (scale points)	30	18.2 ± 4.4 (9.3)	20.4 ± 4.5 (11.8)	0.708 (96.3%)	.001	.005	26.7% (26.7%)
5STS (seconds)	32	19.3 ± 13.5 (13.6)	14.7 ± 5.6 (13.0)	0.462 (71.6%)	.002	.005	43.8% (40.6%)
TUG (seconds)	30	13.5 ± 10.2 (17.6)	11.1 ± 6.7 (9.9)	0.260 (28.0%)	.002	.005	16.7% (-)
TUGcog (seconds)	34	19.7 ± 13.8 (15.2)	16.1 ± 7.2 (14.9)	0.310 (41.9%)	.038	.049	26.5% (-)
PGS (meters/second)	32	0.90 ± 0.27 (11.1)	1.00 ± 0.27 (14.8)	0.549 (85.3%)	.003	.005	37.5% (43.8%)
FGS (meters/second)	32	1.31 ± 0.30 (8.9)	1.43 ± 0.37 (15.8)	0.599 (90.7%)	.002	.005	28.1% (-)
6MWT (meters)	24	347.7 ± 105.2 (6.0)	381.1 ± 120.6 (6.0)	0.539 (70.4%)	.016	.024	41.7% (41.7%)
FFABQmod (scale points)	26	16.3 ± 11.8 (10.5)	15.7 ± 12.0 (6.8)	0.085 (7.0%)	.670	.670	0% (-)

**Table 6:** Pre- and posttest scores and ranks across the assessment battery for individuals diagnosed with mild cognitive impairment with Wilcoxon signed-rank *p* value and Benjamini-Hochberg adjusted *p* value.

	*N* = 37	Pre-mean (mean rank)	Post-mean (mean rank)	Effect size (power)	*p* value	Adjusted *p* value	% improved beyond MDC (MCID)
MoCA (scale points)	37	22.4 ± 4.2 (10.1)	23.8 ± 4.1 (11.9)	0.434 (72.9%)	.013	.017	29.7% (29.7%)
MBT (scale points)	33	19.9 ± 4.2 (6.4)	21.9 ± 3.4 (13.2)	0.693 (97.1%)	.001	.005	27.3% (27.3%)
5STS (seconds)	33	17.0 ± 9.2 (13.7)	13.7 ± 5.1 (7.8)	0.471 (74.7%)	.002	.006	30.3% (27.3%)
TUG (seconds)	37	12.4 ± 7.5 (17.3)	10.7 ± 6.8 (10.9)	0.581 (93.0%)	<.001	.005	8.1% (-)
TUGcog (seconds)	36	17.8 ± 12.6 (16.5)	14.8 ± 14.6 (12.3)	0.365 (56.7%)	.003	.007	25.0% (-)
PGS (meters/second)	34	0.95 ± 0.30 (9.2)	1.01 ± 0.30 (16.4)	0.486 (78.5%)	.011	.017	35.3% (44.1%)
FGS (meters/second)	33	1.31 ± 0.37 (11.0)	1.40 ± 0.40 (13.6)	0.482 (76.6%)	.009	.016	24.2% (-)
6MWT (meters)	29	341.1 ± 129.7 (5.3)	360.9 ± 130.2 (8.1)	0.475 (69.5%)	.022	.025	31.0% (31.0%)
FFABQmod (scale points)	32	15.2 ± 12.6 (11.0)	13.4 ± 12.0 (8.6)	0.197 (19.1%)	.295	.295	6.3% (6.3%)

## Data Availability

Data are available upon reasonable request of the corresponding author.
